# Changes in Gut Microbial Ecology and Immunological Responses of Mice Fed the Insoluble Fraction of *Brassica rapa* L. that was Fermented or Not

**DOI:** 10.1264/jsme2.ME17059

**Published:** 2017-09-27

**Authors:** Sachi Tanaka, Kana Yamamoto, Chisato Hamajima, Fuka Takahashi, Kazuki Yamada, Kanon Furuya, Yutaka Uyeno

**Affiliations:** 1 Academic Assembly (Institute of Agriculture), Shinshu University Minamiminowa, Nagano 399–4598 Japan; 2 Graduate School of Science and Technology, Shinshu University Minamiminowa, Nagano 399–4598 Japan; 3 Supramolecular Complexes Unit, Research Center for Fungal and Microbial Dynamism, Shinshu University Minamiminowa, Nagano 399–4598 Japan

**Keywords:** butyrate-producing bacteria, dietary fiber, fermented vegetable, regulatory T cells

## Abstract

We aimed to investigate the effects of feeding fermented *Brassica rapa* L. on ecological and immunological changes in the mouse gut using *in vitro* cultivation tests and *in vivo* experiments in normal mice. In the preliminary *in vitro* study, two *B. rapa* L. products from different fermentation periods (one d [SF] or six months [LF]) were evaluated along with non-fermented vegetables (NF). Among the components of each product, the insoluble fraction resulted in the most prominent change such as a relative increase in butyrate production during a cultivation inoculated with mouse cecum contents. Based on this result, the boiled water-insoluble fractions of *B. rapa* L. (SF, LF, and NF samples) were selected as test materials for the subsequent *in vivo* experiment. Male C57BL/6J mice were divided into four groups and fed either a control diet (CON) or control diet plus one of the insoluble fractions for two weeks. The NF and LF groups had higher relative populations of *Faecalibacterium prausnitzii* than the CON group. Therefore, colonic butyrate concentrations were higher in the NF and LF groups than in the CON group. The oral administration of *B. rapa* L. extract induced immune regulatory effects, even when mice were fed NF and SF, but not LF, as assessed by an increase in regulatory T cell numbers. Our results indicate that feeding a purified insoluble fraction from *B. rapa* L. affects enteric short-chain fatty acid production and immunological responses in the mouse gut in a similar manner, regardless of the fermentation status.

Vegetable dietary fiber is considered to act as an effective prebiotic and is consumed by various members of the gut microbial community because mammals lack the necessary functions to digest these molecules. The profiles of dominant species in the human gut microbiota may be modified by the intake of dietary fiber (5, 13, 26); these changes may affect the health of an individual by altering metabolic pathways. Among fiber-rich vegetables, those belonging to the *Brassica* genus (the *Brassicaceae* family, *e.g.*, kale, cabbage, broccoli, and turnips) contain a number of fibrous nutrients, polyphenols, and sulfur-containing compounds (12), which mediate the health-promoting properties of vegetables, such as anticancer effects (18), as well as their effects on cholesterol metabolism (1). We previously performed an *in vivo* experiment to examine the effects of feeding the boiled water-insoluble fraction of *Brassica rapa* L. (nozawana) on immune responses through compositional changes in enteric bacteria and short chain fatty acid (SCFA) production in normal mice (30). The findings obtained implied that the generation of butyrate derived from food components rich in dietary fiber was increased, suppressing gut immune functions, such as the induction of regulatory T cells (Tregs); this process may be mediated by alterations in the microbiota, prompting a population switch in *Bacteroidetes* to butyrate-producing bacteria.

*Brassica rapa* L., which is a traditionally and regionally planted vegetable in Japan, is often consumed as a fermented vegetable; during fermentation, indigenous lactic acid bacteria attach to the leaves and stems, resulting in the decomposition of dietary fiber (15). The degree of functionality of butyrate-producing bacteria inhabiting the animal gut may depend on the nature and processing of the fiber fraction. Thus, further studies are required in order to clarify whether changes in the components of the insoluble fraction of *Brassica rapa* L. are caused by the effects of vegetable fermentation on butyrate producers and other members of the gut microbiota. In this study, we aimed to elucidate the effects of the fermentation of *Brassica rapa* L. on ecological and immunological changes in the mouse gut using an *in vitro* cultivation study followed by an *in vivo* study.

## Materials and Methods

### *In vitro* experiment

We obtained two fermentation vegetable products that were prepared via different fermentation periods at room temperature; one was prepared by fermentation for 1 d (short fermentation, SF) and the other by fermentation for 6 months (long fermentation, LF). We also evaluated fresh *Brassica rapa* L. vegetable (no fermentation, NF). These materials were dried at 105°C for 2 h in order to assess the dry matter content (Table 1). Ten grams of each material was soaked in deionized water filled to 100 mL, and samples were then mixed well with gentle stirring at 25°C for 1 h. We measured pH with a glass electrode. Soaked materials were pasteurized at 95°C for 10 min, followed by centrifugation at 10,630×*g* for 10 min to separate solid and liquid fractions. After collecting the liquid fraction, which was used for *in vitro* testing, the resulting suspensions were washed with deionized water twice and dried at 60°C for 16 h. The yields of crude solids are shown in Table 1. We used 133 (NF), 136 (SF), and 167 mg (LF) of crude solids for a one-bottle incubation; each of these was equivalent to 1 g of original materials. All samples (crude liquids, crude solids, and chopped original vegetables) were stored at –20°C until used in the *in vitro* culture tests.

As previously described by Ze *et al.* (36), we used a modified YCFA medium consisting of (100 mL^−1^): casitone (0.25 g), yeast extract (0.1 g), glucose (0.2 g), NaHCO_3_ (0.4 g), cysteine (0.1 g), K_2_HPO_4_ (0.045 g), KH_2_PO_4_ (0.045 g), NaCl (0.09 g), (NH_4_)_2_SO_4 (_0.09 g), MgSO_4_.7H_2_O (0.009 g), CaCl_2_ (0.009 g), resazurin (0.1 mg), and hemin (1 mg). In addition, the following SCFAs were included (final concentrations): acetate (5 mM), propionate (3 mM), iso-butyrate (1 mM), iso-valerate (1 mM), and valerate (1 mM). Cysteine was added to the medium following boiling, and samples (40 mL) were then dispensed into 120-mL glass tubes that were subsequently flushed with CO_2_. After autoclaving, a filter (0.45 μm)-sterilized vitamin solution was added to give the following final concentrations (100 mL^−1^): biotin (1 μg), cobalamin (1 μg), thiamine (1 μg), riboflavin (1 μg), *p*-aminobenzoic acid (3 μg), folic acid (5 μg), and pyridoxamine (15 μg). One gram of fresh vegetable, 10 mL of crude liquid, or defined amounts of crude solids were added to the tubes, followed by flushing with CO_2_, and the tubes were then tightly sealed with rubber caps and an aluminum ring. The inoculants for the cultivation tests were obtained from the cecal samples of four healthy male C57BL/6 mice (6 weeks old). Mice were housed and cared for according to the Guide for the Care and Use of Experimental Animals of Shinshu University. Collected cecal samples (approximately 100 mg mouse^−1^) were immediately suspended together in 4 mL phosphate-buffered saline (PBS), followed by N_2_ flushing, and then used as inoculants (100 μL tube^−1^; syringe injection) within 2 h of collection. Tubes were incubated in an incubator with gentle shaking at 37°C. Three tubes for each experimental compartment were applied for cultivation. Another three bottles without a *Brassica rapa* L. compartment, but with cecum dilution were used as the control (CON). In addition, extra bottles with 1 g of fresh or fermented *Brassica rapa* L. vegetables, but without the cecum inoculation were prepared (three bottles for each material) and cultivated under the same conditions in order to evaluate whether bacteria that naturally adhered to the vegetables affected butyrate fermentation. After a 24-h incubation, the contents of each vial were transferred to centrifuge tubes, centrifuged at 1,000×*g* at 4°C for 10 min, and subjected to measurements as described below.

### Animal experiments and tissue sampling

The preparation of purified insoluble components from the three materials (*i.e.*, NF, SF, and LF) for *in vivo* experiments was performed as previously described (30). Briefly, soaked materials in pure water were autoclaved and homogenized. The resulting suspensions were centrifuged at 2,215×*g* for 10 min to remove large residues. The supernatants were again centrifuged at 20,630×*g* for 5 min, and the pellets were then lyophilized in a freeze-dryer and used as extracts. Samples of 1 g of fresh *Brassica rapa* L. yielded approximately 0.3 mg of residual extract. The sugar compositions of each extract were assessed according to the methods described in a previous study (35).

Animal experiments were also conducted according to our previous study (30). Required approval for the experiments was obtained from the Committee for Animal Experiments of Shinshu University. Due to limitations in the capacity of the facility and animal management, the data presented in this study were obtained over the course of two feeding trials with exactly the same design (20 animals for each trial). Five-week-old male C57BL/6 mice were housed in a specific pathogen-free facility. The standard diet (67% carbohydrate, 19% protein, 4% fat, and 4% ash) consisted mainly of casein (190 g kg^−1^), corn starch (300 g kg^−1^), sucrose (330 g kg^−1^), cellulose (47 g kg^−1^), soybean oil (22 g kg^−1^), lard (18 g kg^−1^), vitamins, and minerals. After a 1-week acclimatization period on the control diet, animals were split into four groups. All groups were fed the same standard diet, and each group were orally administered either the extract of *Brassica rapa* L. (NF, SF, and LF groups) or water (control [CON] group) once daily using an oral sonde for 2 weeks. Each extract was resuspended to 2 mg mL^−1^ in water and provided at 20 mg kg^−1^ body weight (BW) d^−1^. Feed and water were supplied *ad libitum*. BW was measured once daily during this feeding period. All animals completed the experiment without health impairments. Mice were sacrificed by cervical dislocation, and tissues, including the colon, cecum, and spleen, were collected and weighed. After measurements, colonic contents (~0.10 g) were subsampled in 1 mL PBS and mixed thoroughly to equalize their distribution in the buffer. The sampling position of colonic contents from each mouse was unified to the middle part of the colon.

### Chemical and microbial analyses

*In vitro* cultivation samples and colonic samples obtained from *in vivo* tests were subjected to chemical and microbial analyses. An rRNA-based, microbial proportion analysis was conducted by means of the extraction of total RNA using an RNeasy Mini Kit (Qiagen, Valencia, CA, USA) followed by sequence-specific rRNA cleavage (32–34). Briefly, a mixture comprised of the RNA solution, hybridization buffer (375 mM Tris-HCl [pH 7.5], 15 mM EDTA, and 375 mM NaCl), oligonucleotide probe solution, a defined amount of formamide, and RNase-free H_2_O was subsequently heated at 95°C for 1 min to unfold RNA molecules. An enzyme solution (25 mM Tris-HCl, 40 mM MgCl_2_, 25 mM NaCl, 4 mM dithiothreitol, 120 mg bovine serum albumin mL^−1^, and 20 U of cloned Ribonuclease H from *Escherichia coli* [Takara, Otsu, Japan] mL^−1^) was then added to the mixture in order to initiate the cleavage reaction. After an incubation at 50°C for 15 min, a stop solution containing EDTA and sodium acetate was added to the mixture to terminate the reaction. RNA in the mixture was purified by ethanol precipitation and subjected to electrophoresis by a MultiNA Bioanalyzer (Shimadzu, Kyoto, Japan). The signal intensities of the respective peaks in the electropherograms were assessed and converted to peak areas in order to calculate the SSU rRNA population of the target group in total SSU rRNAs. Regarding the detection and quantification of the respective bacterial groups, the following probes were used: Bac303m (*Bacteroides* and *Prevotella*); Erec482m (*Blautia coccoides*-*Eubacterium rectale* group); Rfla1269 (*Ruminococcus flavefaciens*), Rbro730m (*Ruminococcus bromii*), Rrec584 (*Roseburia* subcluster), and Fprau645 (*Faecalibacterium prausnitzii*). These probes were separately applied using the same reaction conditions as those in previous studies (32–34). Quantitative real-time PCR was performed to analyze total bacterial DNA based on a previous study (27) with slight modifications. Bacterial genomic DNAs were extracted from colonic samples using a QIAmp DNA Stool Mini Kit (Qiagen) according to the manufacturer’s instructions. Solutions of extracted DNA were stored at –80°C until used. The primer set for real-time PCR was UniF (GTGSTGCAYGGYYGTCGTCA) and UniR (ACGTCRTCCMCNCCTTCCTC) (9). A CFX96 Real-Time system (Bio-Rad, Hercules, CA, USA) and SYBR Premix Ex Taq Kit (Takara) were applied for real-time PCR. The cycling conditions were initial denaturation at 95°C for 10 s and 40 cycles at 95°C for 5 s and 62°C for 30 s, followed by a melting curve analysis to confirm that the expected PCR products were obtained. The supernatants of suspensions of *in vitro* cultivation samples and colonic content samples were used to analyze organic acids with a high-performance liquid chromatography system, as described previously (34). Since *in vitro* cultivation medium and *Brassica rapa* L. materials contained small amounts of organic acids, we evaluated the true production mass of organic acids during cultivation by subtracting organic acids contained in bottles prior to cultivation. De Man, Rogosa, and Sharpe agar plates (Oxoid, Hampshire, UK) were used for the plate-count enumeration of *Lactobacillus* (incubation at 37°C for 3 d).

### Immunological analyses

A flow cytometric analysis of cultured splenic cells and Tregs staining were conducted as described in our previous study (30). Briefly, spleens were removed from mice in all groups, and single-cell suspensions were passed through 40-μm cell strainers (BD Falcon, Franklin Lakes, NJ, USA). In order to deplete red blood cells, spleen cells were treated with 0.17 M Tris-HCl buffer (pH 7.65) containing 0.83% NH_4_Cl.

In the analysis of cell-surface molecules, we used fluorescein isothiocyanate (FITC)-conjugated anti-CD4 (GK1.5), phycoerythrin (PE)-conjugated anti-CD8 (53-6.7), PE-conjugated CD11b (M1/70), allophycocyanin (APC)-conjugated CD69 (H1.2F3), APC-conjugated CD11c (HL3), and 7-amino-actinomycin D (7-AAD). These antibodies were purchased from BioLegend (San Diego, CA, USA). Spleen cells were stained with fluorescence-labeled monoclonal antibodies (mAbs) and 7-AAD. Expression levels were evaluated by flow cytometry (FACSCalibur, Becton Dickinson, San Jose, CA, USA). Regarding the staining of Tregs, after staining with FITC-conjugated anti-CD4 and APC-conjugated anti-CD25 mAbs (clone: PC61; BioLegend), cells were fixed and permeabilized with a FlowX FoxP3 Fixation & Permeabilization Buffer Kit (R&D Systems, Minneapolis, MN, USA). Permeabilized cells were stained with PE-conjugated anti-mouse Foxp3 mAbs (clone: 150D; BioLegend). Stained cells were subsequently analyzed by flow cytometry (FACSCalibur, Becton Dickinson).

### Statistical analyses

Measurements were analyzed by a one-way analysis of variance with STATA 13.1 (Stata, College Station, TX, USA), followed by Tukey’s multiple comparison test if there was any significance. Differences with *P* values of less than 0.05 were considered to be significant.

## Results

### *In vitro* experiment

**Fig. 1:**
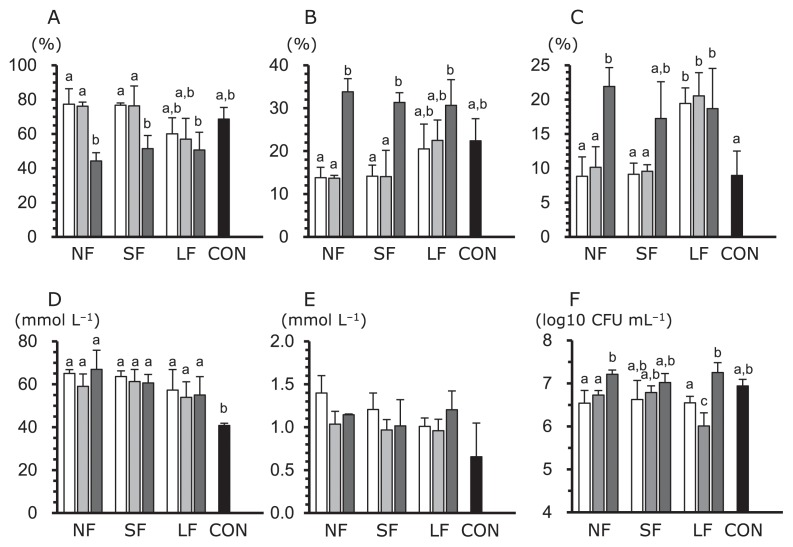
Short-chain fatty acid (SFCA) profiles of and *Lactobacillus* numbers in *in vitro* cultures containing *B. rapa* L. crude fractions inoculated with mouse cecum dilutions. Acetate (A), propionate (B), butyrate (C), total SCFAs (D), lactate (E), and *Lactobacillus* counts (F). NF, not fermented, fresh vegetables; SF, short fermentation (1 d); LF, long fermentation (6 months); CON, control (no *B. rapa* L. fraction). Acetate, propionate, and butyrate are shown as proportions relative to total SCFAs. Bars in the NF, SF, and LF groups indicate raw materials (blank bar), crude fluids (light gray bar), and crude solids (dark gray bar), respectively. Error bars indicate standard deviations (SDs), and bars with different superscripts indicate significant differences (*P*<0.05).

In tubes with mouse cecum contents, total SCFA concentrations did not significantly differ in any of the groups examined, except for the CON group. Additionally, in the NF and SF groups, similar results were observed in cultures treated with the crude solid fraction compared with those in cultures treated with the original material or the crude liquid fraction; lower acetate concentrations, higher propionate concentrations, and higher butyrate concentrations in the crude solid fraction (Fig. 1A, B, C and D). *Lactobacillus* was detected in all groups; however, lactate concentrations were lower than 2 mM in all groups (Fig. 1E and F). The pH values of the solutions ranged between 6.1 and 6.3, possibly due to the buffering effects of sodium bicarbonate, which was added to the cultivation medium (data not shown). In tubes without inoculants, similar results were observed among the three groups, including high lactate concentrations (16.5–25.8 mM) and acetate concentrations (3.9–9.5 mM), but low propionate and butyrate concentrations (less than 1 mM).

### *In vivo* experiment

The sugar proportions of insoluble fractions in each material applied to the *in vivo* experiment were shown in Table 1. The fraction generated from NF and LF exhibited similar sugar profiles, whereas SF contained a markedly higher glucose content than the others. The BWs and organ weights of mice are shown in Table 2. BWs did not significantly differ among the groups at any time point during feeding; thus, mice in each treatment group were considered to grow normally. Neither cecum weights nor colon weights differed among groups.

**Fig. 2:**
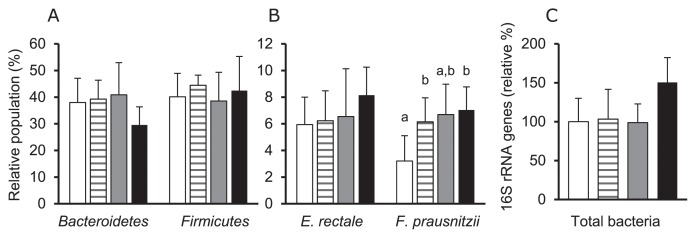
Effects of the oral administration of extracts of *B. rapa* L. on colonic bacterial communities in mice. Relative bacterial populations at the phylum level (*Bacteroidetes*: *Bacteroides* and *Prevotella*; *Firmicutes*: *Blautia coccoides* to the *Eubacterium rectale* group and *Clostridium leptum* subgroup) (A). Relative bacterial populations in the representative butyrate-producing bacteria (*E. rectale* and *F. prausnitzii*) (B). Data shown in Fig. 2A and B were obtained using the sequence-specific rRNA cleavage method. Total bacterial 16S rRNA copies in colonic samples (C). Data are expressed as relative gene copy numbers per colonic contents assuming the average of the control group as 100. Error bars indicate SDs for all mice used in two independent experiments performed together (*n*=10 for all groups). The significance of differences among the CON (blank bar), NF (stripe bar), SF (gray bar), and LF (black bar) groups is indicated by different superscripts (*P*<0.05).

**Fig. 3:**
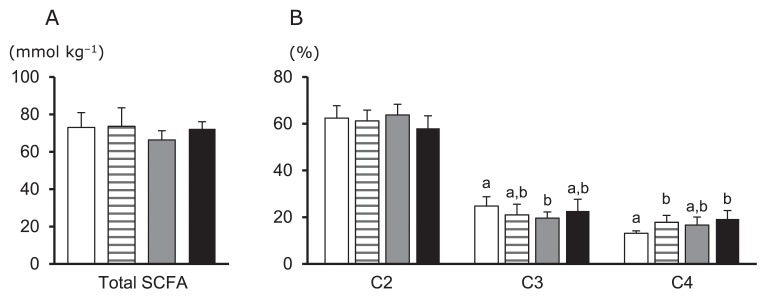
Effects of the oral administration of extracts of *B. rapa* L. on total SCFA concentrations in colon samples (A), and relative molar proportions of acetate (C2), propionate (C3), and butyrate (C4) (B). Data are represented in the same manner as described in Fig. 2.

Bacterial profiles in the colonic contents of mice are shown in Fig. 2. At the phylum level, *Bacteroidetes* (*Bacteroides* and *Prevotella*, assessed by the Bac303m probe) were slightly lower in the LF group than in the CON group (*P*=0.065, Fig. 2A). Moreover, NF and LF groups had higher relative populations of *F. prausnitzii* than the CON group; however, no significant difference was found in relation to the relative population of *E. rectale* (*P*=0.085, Fig. 2B). Total bacterial numbers were not significantly different among groups, but appeared to be slightly higher in the LF group than in the other groups (*P*=0.090, Fig. 2C). The total SCFA levels of the colonic contents did not significantly differ among groups (Fig. 3A). Moreover, regarding the proportions of different SCFAs, that of butyrate was higher in the NF and LF groups than in the CON group, whereas that of propionate was lower in the SF group than in the CON group (Fig. 3B). Lactate and valerate were minor constituents (<0.5 mmol kg^−1^ sample) of the samples.

**Fig. 4:**
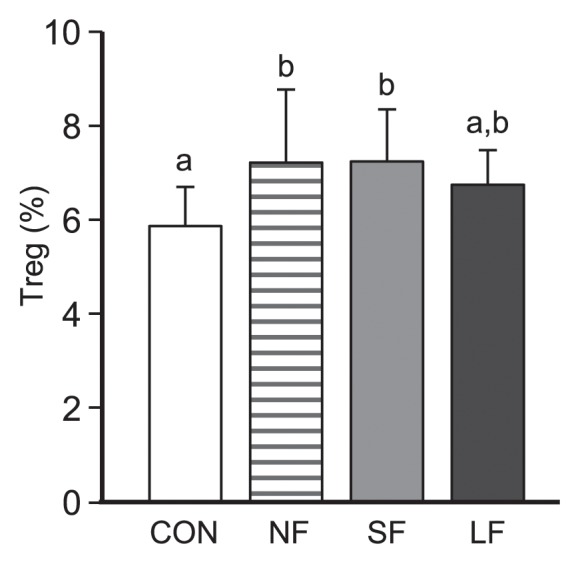
Effects of the oral administration of extracts of *B. rapa* L. on splenic immunological responses. Proportions of Tregs in total spleen cells are displayed. Data are presented in the same manner as in Fig. 2.

There were no significant differences among groups in terms of CD69 expression levels for CD4^+^ or CD8^+^ T cells; however, CD69 expression on CD11b^+^ cells was significantly weaker in the LF group than in the NF group. In addition, CD69 expression on CD11c^+^ cells was weaker in the LF group than in the CON group (data not shown). In contrast, the proportion of Tregs slightly increased in mice orally administered either NF or SF, but not LF (Fig. 4). These results suggested that the oral administration of *Brassica rapa* L. extract induced immunoregulatory effects, even when mice were fed extracts that had fermented for a few d, but not LF extracts, as demonstrated by the increase in Tregs.

## Discussion

In the present study, we examined whether the fermentation of the *Brassica rapa* L. product affected enteric fermentation. According to our *in vitro* results, supplementation with the crude insoluble fraction resulted in a relative increase in butyrate production during cultivation, particularly in the NF and SF groups, which was consistent with the findings from our previous *in vivo* trial (30). In the NF and SF groups, similar results were obtained in cultures treated with the original material or crude liquid fraction, including higher acetate concentrations and lower propionate concentrations. We assumed that readily fermenting carbohydrates (*i.e.*, non-structured carbohydrates [NSCs]) remained in these fractions, which are used preferentially over structured carbohydrates by inoculated bacteria, resulting in differential fermentation profiles from those of the insoluble compartment. Due to the reduced NSC consumed during longer vegetable fermentation periods, compositional differences may become smaller among these components (original material and liquid fraction) in the LF group, which resulted in similar *in vitro* fermentation patterns. Furthermore, according to cultivation results for tubes that were not inoculated, it is also possible that a certain amount of lactate-fermenting bacteria (homofermentative, heterofermentative, or both) (15) was present in fermented *Brassica rapa* L.; however, there was an insufficient number of butyrate-producing bacteria to contribute to the fermentation of vegetables or subsequent *in vitro* cultivation. Based on these results, we hypothesized that insoluble carbohydrates were major components affecting gut fermentation and immunological responses thereafter, while differences in the fermentation periods were not necessarily reflected by *in vitro* cultivation characteristics when insoluble fractions were applied. Thus, subsequent *in vivo* feeding experiments were conducted using insoluble fractions obtained by the same procedure as that of our previous study.

Beyond simply acting as a crude caloric source, butyrate is naturally present at high concentrations in the lumen of the large intestine and is considered to be essential for colonocyte health. Moreover, butyrate may be involved in the prevention and treatment of some chronic digestive diseases (23) and in driving the immune system (19, 20, 24, 29). Our previous findings suggested that increases in copy numbers in the butyryl-CoA:acetate CoA-transferase gene in the *Brassica rapa* L. group were associated with higher concentrations of typical secondary metabolites, such as propionate and butyrate, which are generated from acetate. In the present *in vivo* study, we examined whether the fermentation of *Brassica rapa* L. changed gut fermentation by indigenous bacteria. The utilization of a wide range of polysaccharides is supported by different groups in the butyrate-producing *Firmicutes F. prausnitzii*, the *Clostridium leptum* (or Clostridial cluster IV) cluster, and *E. rectale*/*Roseburia* spp. in the *B. coccoides* (Clostridial cluster XIVa) cluster (3, 4, 7, 17). Interestingly, one butyrate-producing group (*Faecalibacter*) was increased in colonic samples of the NF and LF groups in our *in vivo* test, in which this group was found to utilize a wide range of carbohydrates, such as mono- or disaccharides and long-chain inulin; however, acetate was the main substrate for energy acquisition (6, 21). Therefore, this group may have used either increased available carbohydrates generated from *Brassica rapa* L. fermentation for a longer period or acetate as a major product of other fibrolytic bacterial groups in the colon (16). In relation to this finding, we previously investigated the effects of consuming a cultivar of *Brassica oleracea* (kale) on mouse intestinal ecology and find significant changes in colonic butyrate production in accordance with the proportional shift observed in the microbiota towards the *B. coccoides* cluster (34). Therefore, although the underlying mechanisms may be different, a wide range of *Brassica* family vegetables presumably possess modulating functions for the gut microbiota.

According to the less distinctive results among insoluble fractions of NF, SF, and LF groups in the *in vitro* test, we did not assume to find a prominent difference in fermentation characteristics in the *in vivo* test. However, regarding the insoluble fraction generated from SF samples in our *in vivo* test, NF and LF samples had markedly different effects on gut communities. These differences may be dependent on the varying kinetics of carbohydrate utilization, given that there were only slight differences in the proportions of sugars in crude SF solids from those of the other two groups. This result may be attributed to the fibrous part of *Brassica rapa* L. being partly decomposed following very short fermentation to generate a fragile structure, which may be easily broken by weak acids. However, this decomposition may have resulted in the release of soluble sugars that were consumed during a longer fermentation. This is based on compositional characteristics indicated by the relatively higher glucose content found in the purified insoluble fraction of SF, whereas a lower glucose content was found in NF and LF. Otherwise, during the *in vivo* test period, the gut flora may have been adjusted to similar proportions, regardless of fiber characteristics (*i.e.*, average molecular weight or modifications in sugar chains). Even NF and LF exerted similar effects; however, these two fractions may be obtained from different amounts of each original material. Based on our results, LF was expected to provide a greater amount of the insoluble fraction than NF, which may have been more effective if it was tested based on the weight of the original material.

The contributions of the gut microbiota to the development of the immune system have been extensively characterized (2, 14, 29). Previous studies demonstrated that the stronger butyrate-induced expression of IL-10, a modulator of Treg function, may be crucial for obesity-associated and systemic inflammation (20, 22, 28). Moreover, butyrate decreases the intestinal expression levels of tumor necrosis factor-α, IL-1b, and IL-6 in patients with Crohn’s disease (11). Our results showed that feeding mice the insoluble fraction significantly increased the levels of the anti-inflammatory cytokine IL-10 and slightly increased splenic Treg numbers, but significantly reduced the population of cells expressing activation markers, both of which may support the role of the microbiota in the development of the mucosal adaptive immune system (30). Therefore, we consequently aimed to identify changes in colonic immune function by supplementing animals with different compositions of fibrous fractions, potentially through the production of SCFAs derived from anaerobic microbial respiration in the gut. These results supported the positive findings of previous experiments with regards to immunological suppression, particularly for the enhanced expression of Tregs in mice fed the insoluble fraction of *Brassica rapa* L. Commensal fiber-fermenting bacteria are regarded as a driver for T-regulatory lymphocyte development (10, 20), thereby stimulating colonocyte immunogenicity. Our preliminary *in vitro* assessment showed that *Brassica rapa* L. may inherently possess components that directly affect the systematic immune response unrelated to enhanced butyrate production, and vegetable fermentation appeared to enhance immune effects (unpublished data). Other components such as plant polyphenols are also known to contribute to the prevention of inflammatory and autoimmune diseases because we revealed a new mechanism through which procyanidin B2 gallates inhibit interferon-γ and interleukin-17 production (31). The nature and processing of vegetable-derived dietary fibers may also be responsible for exerting direct and indirect effects on host immunological functionality because the present *in vivo* experiment showed that the fermentation of the *Brassica rapa* L. vegetable did not necessarily enhance the Treg response.

## Conclusion

In the present study, we analyzed the effects of *Brassica rapa* L. fermentation on the nature of gut-simulating cultivation and found that SCFA proportions in cultures were highly dependent on the degree of fermentation of vegetables and the fractions separated based on solubility in water. In contrast, feeding mice a purified insoluble fraction from *Brassica rapa* L. affected enteric SCFA production and immunological responses in the gut in a similar manner, regardless of fermentation, when supplied at the same amount. These results imply that the expansion of the effects of dietary fiber in fermented vegetables on the structure and function of the animal gut depend on the amount of the product used compared with that of fresh (non-fermented) vegetables. Alternatively, these components may affect the enteric microbiota more prominently when applied to particular conditions related to nutritional disorders, such as diabetes or obesity (8, 20, 25).

## Figures and Tables

**Table 1:** Dry matter and pH of tested materials, crude solid yields (for in vitro tests), and sugar proportions of insoluble fractions (for in vivo tests) used in this study.

	NF	SF	LF
Dry matter (g kg^−1^)	61.5	62.6	70.6
pH	5.9	5.16	5.38
Crude solid yield (for the *in vitro* test, mg g^−1^ material)	13.3	13.6	16.7
Sugar proportions of purified insoluble fractions (for the in vivo test, mg g^−1^ fraction)			
Arabinose	12.3	7	8.7
Glucose	28.7	271.3	48.2
Galactose	16.8	15.6	19.7
Mannose	3.8	2.6	2.7
Rhamnose	1	ND	0.9
Xylose	2.1	2.3	3.6

NF, no fermentation B. rapa L. group; SF, short fermentation *B. rapa* L. group; LF, long fermentation *B. rapa* L. group. ND, not detected.

**Table 2:** Body weights and organ weights of the cecum and colon in mice orally administered water or *B. rapa* L. extracts fermented for 1 d or 6 months.

	CON	NF	SF	LF
Body weight (g)	20.7±0.8	20.4±1.2	20.4±1.3	20.0±0.8
Cecum (mg)	501.8±6.5	512.5±70.9	469.1±76.5	474.1±30.4
Colon (mg)	303.9±46.4	345.5±31.8	325.3±34.8	343.5±29.0

CON, control group; NF, no fermentation *B. rapa* L. group; SF, short fermentation *B. rapa* L. group; LF, long fermentation *B. rapa* L. group. Values are expressed as means±SEMs.
